# The role of m5C, m1A and m7G modifications in tumors of urinary system

**DOI:** 10.3389/fcell.2025.1549588

**Published:** 2025-07-30

**Authors:** Wei Xue, Yuanyuan Zhao, Gong Zhang, Zhiyuan Li, Jixin Li, Xiang Fei

**Affiliations:** ^1^ Department of Urology, Shengjing Hospital of China Medical University, Shenyang, China; ^2^ Department of Obstetrics and Gynecology, Shengjing Hospital of China Medical University, Shenyang, China

**Keywords:** RNA modification, m5C, m1A, m7G, urological tumours

## Abstract

Malignant tumors of the urinary system, such as kidney cancer, bladder cancer, and prostate cancer, remain a significant challenge despite the various treatment options available. Identifying therapeutic targets for urological tumors is crucial due to the potential for recurrence and metastasis. Recent research has highlighted the importance of RNA modifications in post-transcriptional regulation, impacting various biological functions in urological tumors, including tumorigenesis, progression, metastasis, and drug resistance. However, the specific mechanisms underlying these interactions are not fully understood. This review will focus on exploring the regulatory role of RNA modifications like m1A, m5C, and m7G in urological tumors, shedding light on the pathways and molecular mechanisms involved. This analysis aims to provide new insights for the treatment of urological tumors.

## Introduction

Malignant tumors of the urinary system predominantly consist of kidney cancer, bladder cancer, and prostate cancer (Günther et al., 1970). In 2024, the United States reported 299,010 new cases of prostate cancer, with 35,250 deaths, ranking it as the most common urinary system tumor. Additionally, there were 83,190 new cases of bladder cancer and 81,610 new cases of kidney cancer, with corresponding mortality rates of 12,290 and 9,450, respectively (Siegel et al., 2024). Generally, surgery remains the primary treatment for urinary system tumors, often resulting in a favorable prognosis (Kumar et al., 2018). However, for patients with advanced tumors who are no longer eligible for surgery, identifying the mechanisms influencing tumor progression and discovering new therapeutic targets can significantly enhance patient prognosis.

As research on RNA modification progresses, over 100 types of RNA modifications have been identified (Boccaletto et al., 2022). These modifications target both mRNAs and non-coding RNAs, with ribosomal RNAs and transporter RNAs showing the highest frequency of modifications (Roundtree et al., 2017; Ontiveros et al., 2019; Li and Mason, 2014). The 5′cap structure is ubiquitous in mRNA (nearly 100% of mRNAs have this modification), but the proportion of internal m^7^G (occurring within the chain) is approximately 0.01%–0.1%. It accounts for about 0.1%–0.3% of mRNA modifications, is sparsely distributed, but is more significant in non-coding RNAs (such as tRNA), accounting for about 10%–20% (Sakai et al., 2016). For m1A modification, each rRNA molecule typically has only 1-2 m1A sites, with a modification ratio close to 100%, yet its rate for mRNA modification is less than one percent (Oerum et al., 2017). Furthermore, studies have revealed that m5C occurs at a modification rate of approximately 5%–10% in tRNA, and is generally sparsely distributed in mRNA but enriched in the coding sequence (CDS) and 3′untranslated region (3′UTR), playing a crucial role in regulating mRNA stability and translation efficiency (Sun et al., 2023). In RNA modification, the key enzymes include “writers,” “erasers,” and “readers,” whose corresponding roles are to catalyze the addition of chemical modifications to RNA molecules, remove RNA modifications (reversing the action of writers), and recognize and bind to specific modifications, thereby triggering downstream effects (Haruehanroengra et al., 2020). RNA modifications have diverse molecular functions that can impact RNA shearing, stability, protein translation efficiency, and intermolecular interactions. Aberrant expression and altered activity of RNA-modifying enzymes can lead to diseases such as inflammation and tumors (Chen et al., 2021; Luo et al., 2021).

RNA modifications are prevalent in various types of RNAs, including mRNAs and non-coding RNAs like microRNAs (miRNAs), long non-coding RNAs (lncRNAs), and circular RNAs (circRNAs) (Zhang et al., 2023a; Luo et al., 2023; He et al., 2023). Notably, tRNAs and rRNAs undergo frequent modifications, primarily impacting protein translation efficiency and fidelity enhancement (Agris, 2008). Specifically, rRNA modifications are concentrated at decoding sites, regions near the peptidyl transfer center (PTC), and other functional sites, influencing protein synthesis efficiency (Deca et al., 2002; Natchia et al., 2017). On the other hand, tRNA modifications predominantly affect codons, thereby regulating protein turnover (Chan et al., 2015; Chionh et al., 2016; Gkatza et al., 2019). Fluctuations in the external environment and internal molecular disturbances can disrupt the dynamic equilibrium of RNA modifications, serving as a compensatory mechanism for cells to adapt to environmental changes (Sonenberg and Hinnebusch, 2009; Dominissini et al., 2012; Blanco et al., 2016; Chan et al., 2010). This suggests a potential role of RNA modification in tumor development. In recent years, advancements in RNA modification detection technology have revealed that N1-methyladenosine (m1A), 5-methylcytidine (m5C), and N7-methylguanosine (m7G) can impact tumor proliferation, invasion, migration, and drug resistance (Xue et al., 2023; Wang et al., 2023a; Luo et al., 2022). m5C, m1A, and m7G modifications are important epitranscriptomic modifications that have demonstrated critical roles in various fields such as virology, cancer, developmental biology, and immune regulation in recent years. With the advancement of detection technologies and in-depth functional studies, the dynamic regulatory mechanisms and physiological and pathological functions of these modifications are gradually being revealed. Research has found that m5C modifications are widely present in mRNA, tRNA, rRNA, and viral RNA, and exhibit dynamic changes across different tissues and cell types (Huang et al., 2019a). In tumor immunity, metabolic disorders, and aging-related diseases, m1A modification enzymes (such as TRMT6/TRMT61A, ALKBH3) may serve as potential intervention targets (Miao et al., 2025). m7G modification is highly expressed in various cancers (such as gastric cancer, oral squamous cell carcinoma, colorectal cancer, and thyroid cancer) and promotes tumor proliferation, metastasis, and metabolic reprogramming by regulating key genes (such as SDHAF4, NEK1, ICAM-1, and TNF-α) (Xu et al., 2025; Cai et al., 2025).

This review examines the influence of RNA modifications, specifically m1A, m5C, and m7G, on urological tumors. It delves into the pathways and molecular mechanisms linked to RNA modifications that could impact urological tumors. Additionally, it discusses the significance of RNA-modifying enzymes in urological tumors and their potential as targets for novel treatment approaches.

## 5-methylcytosine

### m5C overview

**FIGURE 1:**
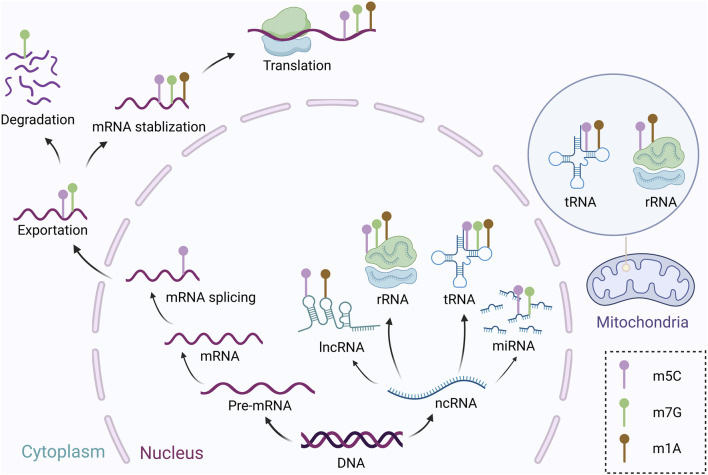
The roles of m5C, m1A, and m7G in cellular processes. Purple stands for m5C, and this modification mainly plays roles in mRNA splicing, exportation, stabilization, and translation, and is also involved in ncRNA—lncRNA, miRNA, tRNA, and rRNA, and in mitochondrial activity, it also affects the function of tRNA and rRNA. Brown represents m1A modification, which is primarily associated with mRNA stabilization and translations, and also affects lncRNA, rRNA, and tRNA in the cell nucleus, as well as rRNA and tRNA in the mitochondria. Green indicates m7G, this modification participates in mRNA exportation, degradation, stabilization, and translation, as well as ncRNAs such as miRNA, rRNA, and tRNA.

The m5C modification is a highly conserved and prevalent RNA modification that can be found on a wide range of RNAs (Figure 1) (Zhao et al., 2024a). For example, m5C modifications are commonly found in the variable loop and anticodon loop of tRNAs, influencing tRNA stability and translation efficiency (Schaefer et al., 2010). In the rRNAs of eukaryotes (such as yeast and humans), m5C modifications are catalyzed by the NSUN methyltransferase family, affecting ribosome assembly and translation fidelity (Zheng et al., 2023a). m5C modifications have been detected in the coding regions and untranslated regions (UTRs) of mRNAs, potentially regulating mRNA stability, nuclear export, and translation efficiency (Yang et al., 2017). A limited number of studies suggest that snoRNAs or precursor miRNAs may possess m5C modifications, though their functions remain unclear (Blanco et al., 2011). Early detection methods for RNA modifications were primarily chromatographic, limiting detection to modifications with high abundance like those found in tRNAs and non-coding RNAs (Boccaletto et al., 2018). However, with the development of purification techniques based on poly-A tails, a variety of modifications on mRNAs, including m6A and m5C, have been discovered (Dubin and Taylor, 1975). Currently, detection of m5C is primarily done using the sodium bisulfite method or immunoprecipitation with m5C-specific antibodies (García-Vílchez et al., 2019). RNA bisulfite sequencing is a valuable tool for quickly and accurately detecting 5-methylcytosine modifications on RNA, particularly for highly expressed RNAs such as tRNAs and rRNAs (Dai et al., 2024; Blanco et al., 2014). Despite its utility, the identification of m5C modification sites remains a topic of debate (Huang et al., 2019a). Research has revealed over 10,275 m5C modification sites on mRNAs and tRNAs in animal cells, with a notable concentration in the untranslated region and Argonaute binding region (Squires et al., 2012). Conversely, some studies suggest that RNA methylation modifications are rare in mouse embryonic stem cells and challenging to detect in mRNAs (Legrand et al., 2017). Recent studies have focused on achieving single-base resolution and quantification of modification scores using biochemical methods to identify RNA modification sites. This approach shows promise for achieving base-resolution localization of low-abundance RNA modifications in mRNAs (Zhang et al., 2024). Therefore, there is still a need for further improvements in the technical methods for sequencing and localizing RNA methylation modifications to achieve more precise single-base region localization.

### Regulation of coding RNAs and non-coding RNAs by m5C

Although m5C is not as widely enriched in RNA modification as m6A, the regulatory role of m5C for both coding and non-coding RNAs has been gradually discovered with the development of epigenetics (Table 1). This modification has been found to affect the biological processes of cells. In thyroid cancer, a high m5C modification state has been observed, leading to increased tRNA stability, intracellular leucine transport, and ultimately enhancing the translation efficiency of certain proto-oncogenes (Li et al., 2023a). Studies have also shown that a decrease in m5C levels of tRNAs can result in tRNAGly coencoder dysfunction and reduced protein translation efficiency (Blaze et al., 2021). M5C modification of tRNA at sites 48, 49, and 50 in mammalian mitochondria enhances tRNA stability and supports phosphonate oxidation during cell differentiation (Van Haute et al., 2019). The tRNAs that undergo m5C modification exhibit enhanced stability and translation efficiency. In the event of DNA damage, there is an upregulation of rRNA and non-coding RNAs, with RNA modification (m5C) contributing to R-loop stability and involvement in transcriptionally linked homologous recombination (Chen et al., 2020a). The smooth assembly of mitochondrial ribosomes in mammals relies on the coordinated maturation of both the large subunit (LSU) and the small subunit (SSU), with 12S rRNA methylation ensuring that only mature LSUs and SSUs can be assembled correctly (Metodiev et al., 2014). This process serves as a crucial factor in ribosome assembly. In cholangiocarcinoma, the functional LncRNA (NKILA) undergoes m5C modification upon interaction with YBX1, leading to its stabilization and promoting tumor progression (Zheng et al., 2022). Similarly, m5C modification enhances circ_0102913 expression, contributing to malignancy in colon cancer through the miR-571/RAC2 axis (Hou et al., 2024). Detecting methylation modifications in less abundant miRNAs poses challenges due to sample purification and the presence of interfering highly abundant RNAs (Richter et al., 2022; Schaefer et al., 2017). Liquid chromatography-tandem mass spectrometry (LC-MS/MS) nucleoside analysis and liquid-solid two-step hybridization (LSTH) were utilized to identify m5C modifications in miR-21-5p, Let-7a/e-5p, and miR-10a-5p, revealing modifications at each site to be less than 1% (Lü et al., 2024). This underscores the need for further development in detection techniques to enhance the sensitivity and specificity for identifying low abundance RNA modifications. For coding RNAs, m5C modifications primarily enhance mRNA stability and protein translation efficiency. YBX1, a reader of m5C, promotes target mRNA stability by recognizing the indole loop of W65 in the cold shock structural domain of the target mRNA, leading to the recruitment of ELAVL1 (Chen et al., 2019a). Additionally, PEBP1P2 interacts directly with 5-methylcytosine (m5C)-containing PEBP1 mRNA and facilitates the stabilization of PEBP1 mRNA by recruiting the YBX1/ELAVL1 complex (Yang et al., 2022a). NSUN6, an m5C writer, has been shown to drive target genes to undergo m5C modifications that impact translation termination, potentially playing a role in quality control of translation termination fidelity (Selmi et al., 2021). However, the precise mechanism remains unclear.

**TABLE 1:** Molecular mechanism and biological functions of RNA modifications.

RNA modifications	Disease type	Biological function	Molecular mechanism	Expression	Ref
m5C	Thyroid cancer	Increase intracellular leucine transport	Stablize tRNA	Upregulated	Yang et al. (2017)
	Depression	Regulate glutamatergic neurotransmission	Reduced protein translation efficiency	Downregulated	Blanco et al. (2011)
	-	Support phosphonate oxidation	Stablize tRNA	Upregulated	Boccaletto et al. (2018)
	-	Mitochondrial ribosome maturation	rRNA assembles	Upregulated	García-Vílchez et al. (2019)
	Cholangiocarcinoma	Promote tumor progression	Stablize LncRNA (NKILA)	Upregulated	Dai et al. (2024)
	Colorectal cancer	Promote proliferation, invasion and migration	Stablize circRNA (circ_0102913)	Upregulated	Blanco et al. (2014)
	Bladder cancer	Promote tumor progression	Stablize mRNA	Upregulated	Zhang et al. (2024)
	Renal cell carcinoma	Promote migration, invasion and metastasis formation	Stablize mRNA (PEBP1)	Downregulated	Li et al. (2023a)
m1A	Hepatocellular carcinoma	Promote cholesterol synthesis	Stablize tRNA, Promote protein translation	Upregulated	Zhu et al. (2022)
	Urothelial carcinoma of the bladder	Participate in the unfolded protein response	Stablize tRNA	-	Alshaker et al. (2019)
	Adoptive transfer colitis model	Inhibit T cell activation	Promote translation efficiency	Downregulated	Kar et al. (2016)
	Yeast	Carbohydrate metabolism, translation, and ribosome synthesis	Maintain rRNA an optimal 60S conformation	-	Wang et al. (2023b)
	*Escherichia coli*	Regulate protein synthesis and cell growth	Maintain 16S rRNA modification	-	Sun et al. (2022)
	-	Inhibit the expression of P53	Mediated 28S rRNA modification	-	Sun et al. (2020b)
	Oplastic transformation of oral Mucosa	Promote MYC and PD-L1 protein synthesis	Stablize tRNA	Upregulated	Zhang et al. (2023b)
	Melanoma	Inhibits the malignant transformation of cancers	Promote mRNA stability and translational efficacy (SP100A)	Downregulated	Dunn (1961)
	Mammalian cells	Promote ciliogenesis in mammalian cells	Inhibits mRNA stability and translational efficacy (Aurora A)	-	Liu et al. (2024a), Zhou et al. (2019)
m7G	Bladder cancer	Promote proliferation, migration and angiogenesis	Promote translation efficiency (VEGFA)	Upregulated	Monshaugen et al. (2024)
	Bladder cancer	Promote proliferation and migration	Promote translation efficiency (TROP2), Stablize miRNA (miR-760)	Upregulated	Shimada et al. (2012), Malbec et al. (2019)
	Lung cancer	Promote proliferation and migration	Promote mRNA translation efficiency	Upregulated	Koike et al. (2012), Ohira and Suzuki (2016)
	Intrahepatic cholangiocarcinoma	Regulate cell cycle, epidermal growth factor receptor and tumor drug resistance	Stablize tRNA, Promote mRNA translation	Upregulated	Ueda et al. (2018), Cheng et al. (2022)
	Head and neck squamous cell carcinoma	Promote tumor progression and metastasis	Promote mRNA translation efficiency	Upregulated	Boulias and Greer (2019), Furuichi (2015)
	Osteosarcoma	Promote proliferation, migration and invasion capacities, Modulate chemotherapy resistance	Promote mRNA translation efficiency, Stablize miRNA (miR-26a-5p)	Upregulated	Zhu et al. (2018), Shoombuatong et al. (2022)
	Acute myeloid leukemia	Promote cell proliferation and inhibit apoptosis	Stablize tRNA	Upregulated	Zhang et al. (2022)
		Regulate cell migration	Disrupt primary miRNA transcript (pri-miRNA)	-	Zhang et al. (2019)

### Effect of m5C modification on the signal transduction pathways

An abnormal increase in NSUN2 in esophageal squamous cell carcinoma was found to promote increased m5C abundance. Additionally, the protein LIN28B was shown to promote GRB2 mRNA stabilisation in an m5C-dependent manner, leading to activation of the PI3K/AKT and ERK/MAPK signalling pathways (Su et al., 2021). Similarly, m5C modifications were found to be significantly higher in hepatocellular carcinomas compared to adjacent normal tissues. Transcriptomic analyses revealed that hypermethylation modifications activated phosphokinase signalling pathways such as the PI3K and Ras pathways (Song et al., 2023; Xiang et al., 2020). According to bioinformatics analysis, DNMT1, the key enzyme of m5C, is closely associated with immune infiltration in liver cancer. The growth factor β (TGF-β)/EMT pathway may be activated to facilitate tumor invasion and metastasis (Gu et al., 2021). In rheumatoid arthritis, NSUN2 is upregulated to drive inflammatory progression through the SFRP1/Wnt/β-catenin signaling axis, a process that can be counteracted by the m5C demethylase FTO (Huang et al., 2024). Following TRDMT1 (m5C methyltransferase) knockout and sequencing of both wild-type and HEK293 cells using RNA-Seq and RNA-bisseq, GO analysis of differentially expressed genes revealed a notable enrichment of the Notch signaling pathway (Xue et al., 2019). In non-small cell lung cancer, LINC02159 binds to the Aly/REF output factor (ALYREF) to promote YAP1 mRNA stability in an m5C-dependent manner, thereby activating Hippo and β-catenin signaling pathways (Yang et al., 2023a). The above explanation indicates that m5C can upregulate numerous signaling pathways, thereby regulating cellular biological functions such as tumor cell proliferation, apoptosis inhibition, invasion, migration, and angiogenesis. Overall, m5C methylation modification is prevalent in various diseases and enhances disease progression by influencing the activity of multiple signaling pathways (Table 2).

**TABLE 2:** RNA modification regulates multiple disease pathways.

RNA modifications	target RNA	Disease type	Pathway	Pathway status	Ref
m5C	mRNA	Esophageal squamous cell carcinoma	PI3K/AKT, ERK/MAPK	Activation	Van Haute et al. (2019)
	mRNA, tRNA, rRNA	Hepatocellular carcinomas	PI3K/AKT, Ras, TGF-β	Activation	Chen et al. (2020a), Metodiev et al. (2014), Zheng et al. (2022)
	mRNA	Rheumatoid arthritis	Wnt/β-catenin	Activation	Hou et al. (2024)
	mRNA	Non-small cell lung cancer	Hippo, β-catenin	Activation	Schaefer et al. (2017)
m1A	tRNA, rRNA	Gastrointestinal tumors, Hepatocellular carcinoma	PI3K/AKT	Activation	Wu et al. (2022), Li et al. (2016)
	tRNA	Advanced hepatocellular carcinoma	Hedgehog	Activation	Chen et al. (2020b)
	rRNA		P53	Inhibition	Sun et al. (2020b)
m7G	mRNA, rRNA	Ameloblastoma, Transplantation of endothelial progenitor cells	MAPK	Activation	Ueda et al. (1991), Zhao et al. (2021)
	tRNA	Pancreatic Cancer	MEK/ERK	Activation	Chen et al. (2023b)
	mRNA, tRNA	Head and neck squamous cell carcinoma	PI3K/AKT/mTOR	Activation	Boulias and Greer (2019)
	tRNA	Nasopharyngeal carcinoma	WNT/β-catenin	Activation	Ma et al. (2021)
	tRNA	Intrahepatic cholangiocarcinoma, hepatocellular carcinoma	EGFR	Activation	Ueda et al. (2018), Cheng et al. (2022)

### The m5C writers

m5C modifying enzymes can be categorized into writers, erasers, and readers, which are responsible for modifying various types of RNA (Table 3). m5C modified writers consist mainly of the NOL1/NOP2/SUN (NSUN) domain protein family, DNA Methyltransferase (DNMT), and TRNA Aspartic Acid Methyltransferase 1 (TRDMT1) (Buj et al., 2004; Motorin et al., 2010). Studies have shown that multiple types of RNA can undergo m5C modification (Figure 1). The main function of m5C is to maintain RNA stability and retard degradation, with S-adenosylmethionine (S-adenosylmethionine) as a methyl donor to form a methylation modification on cytosine. Different cellular regions are mediated by different modifying enzymes, and in the nucleus NSUN2, NSUN5, NSUN6, NSUN7, and NOP2 are responsible for the methylation modification of mRNAs and some non-coding RNAs (Bourgeois et al., 2015; Xu et al., 2020; Heiss et al., 2019; Yang et al., 2021; Aguilo et al., 2016; Surovtseva and Shadel, 2013). In mitochondria, NSUN2, NSUN3, and NSUN4 play a key role in tRNA and 12S rRNA methylation, facilitating mitochondrial ribosome assembly (Nakano et al., 2016; Kawarada et al., 2017; Van Haute et al., 2016; Lenarčič et al., 2021; Cipullo et al., 2021). While some methyltransferases modify the same RNA types, they target different modification sites. For instance, NSUN1/NOP2 and NSUN5 target m5C 4447 and m5C 3782 on 28S rRNA, respectively (Bourgeois et al., 2015; Heiss et al., 2019; Janin et al., 2019). Moreover, NSUN6 and DNMT2 modify m5C72 and m5C38 on tRNA, respectively (Schaefer et al., 2010; Yang et al., 2021; Tuorto et al., 2012; Goll et al., 2006; Shanmugam et al., 2015). NSUN2, the most extensively studied methyltransferase, acts on mRNA (Yang et al., 2017; Xu et al., 2020; Cai et al., 2016; Xing et al., 2015; Li et al., 2017a), tRNA (Van Haute et al., 2019), miRNA (Yuan et al., 2014; Yang et al., 2015), and lncRNA (Sun et al., 2020a; Li et al., 2018). NSUN3 is an intra-mitochondrial methyltransferase that facilitates the S-adenosylmethionine (AdoMet) dependent methylation initiation of mt-tRNA, leading to the biogenesis of 5-formylcytidine (f5C) 34 and ultimately promoting protein synthesis (Nakano et al., 2016; Kawarada et al., 2017; Van Haute et al., 2016). NSUN4 is involved in the m5C modification of rRNA (Metodiev et al., 2014; Lenarčič et al., 2021; Cipullo et al., 2021; Cheng et al., 2021; Yakubovskaya et al., 2012; Cámara et al., 2011) and mRNA (Yang et al., 2022b), which enhances ribosome conformational stability, assembly maturation, and protein translation. In humans, NSUN5 primarily methylates the m5C 3782 site of rRNA, contributing to protein stability (Heiss et al., 2019; Janin et al., 2019). NSUN6 is responsible for modifying the m5C 72 sites of tRNACys/Thr in the cytoplasm (Yang et al., 2021) and methylating certain mRNA 3′UTR regions (Yang et al., 2021). Lastly, NSUN7 is responsible for the m5C modification of enhancer RNAs (eRNAs), promoting eRNA stability and enhancing transcription (Aguilo et al., 2016).

**TABLE 3:** The type of RNA modified by the RNA modifying enzyme corresponds to the modification.

RNA modifications	Enzyme	Target RNA	Refs
m5C, Writer	NOP2	mRNA, rRNA	Yang et al. (2017), Surovtseva and Shadel (2013)
	NSUN2	tRNA, mRNA, LncRNA, rRNA	Yang et al. (2017), Haltom et al. (2014), Schosserer et al. (2015), Frye and Watt (2006)
	NSUN4	rRNA , mRNA	Yang et al. (2017), Metodiev et al. (2014)
	NSUN5	rRNA , mRNA	Yang et al. (2017), Schosserer et al. (2015)
	NSUN6	mRNA, tRNA	Yang et al. (2017), Blanco et al. (2011)
	DNMT2	tRNA	Schaefer et al. (2010)
Eraser	ALKBH1	tRNA, mtRNA	Uno et al. (2013), Cho et al. (2016)
Reader	ALYREF	mRNA	Wang et al. (2023b)
	YBX1	mRNA	Sun et al. (2022)
m1A, Writer	TRMT6/61A	mRNA, tRNA	Suzuki et al. (2009), Xie et al. (2024), Li et al. (2022a), Monshaugen et al. (2024)
	TRMT10C	tRNA	Vilardo et al. (2012)
Reader	YTHDF1	mRNA, tRNA, rRNA, lncRNA	Li et al. (2016), Dominissini et al. (2016)
	YTHDF3	tRNA, mRNA	Suzuki (2021), Dominissini et al. (2016)
Eraser	ALKBH1	rRNA	Ougland et al. (2012)
	ALKBH3	mRNA, tRNA	Liu et al. (2016), Koike et al. (2012), Ueda et al. (2018)
m7G, Writer	METTL1/WDR4	mRNA, miRNA, tRNA	Chen et al. (2023b), García-Vílchez et al. (2023), Xie et al. (2022), Ying et al. (2021), Zhang et al. (2023c)
	WBSCR22	rRNA	van Tran et al. (2019)
	TRMT112	tRNA	Alexandrov et al. (2002)
Reader	eIF4E1	mRNA	Sonenberg and Hinnebusch (2009)

### The m5C erasers

While m5C writers have been extensively studied, research on m5C erasers has been more limited. Current research indicates that m5C erasers primarily consist of Tet family (Tet1-3) members and ALKBH1. Tet family enzymes not only facilitate DNA methylation modifications, but also catalyze 5-hydroxymethylcytosine (5-hmrC) activity in RNA (Fu et al., 2014). In mammals, RNA m5C is oxidized to produce 5-hydroxymethylcytidine and 5-formylcytidine (Huber et al., 2015). Tet1 plays a role in clearing m5C modifications at hybrid sites during DNA damage repair, thereby promoting homologous recombination (Yang et al., 2022c). Furthermore, Tet1 has been shown to decrease the m5C levels of RelB mRNA and stabilize its mRNA expression (Lin et al., 2024). Tet2 has been found to significantly reduce tRNA m5C levels *in vitro*, leading to enhanced translation levels (Shen et al., 2021). Alpha-ketoglutarate-dependent dioxygenase homologue 1 (ALKBH1), primarily involved in demethylation, exhibits substrate diversity and suggests a diverse biological function (Zhang and Wang, 2021).

### The m5C readers

As research progressed, various m5C-related readers were gradually identified, including ALYREF, YBX1, LIN28B, YTHDF2, RAD52, and FMRP proteins. The first m5C reader identified was Aly/REF export factor (ALYREF) (Yang et al., 2017), known for its role in promoting mRNA stability and export, closely linked to amino acid metabolism and immune regulation (Meng et al., 2024; Nulali et al., 2024; Eckwahl et al., 2020). YBX1, another well-studied m5C reader, primarily utilizes the cold shock domain (CSD) to bind to target mRNA and facilitate m5C modification (Su et al., 2021; Chen et al., 2019c; Yang et al., 2019). YBX1 functions as an RNA-binding protein, interacting with target RNA to enhance stability and translation, influencing biological processes such as cell autophagy, lipid synthesis (Wang et al., 2023a; Wu et al., 2023), ferroptosis (Chen et al., 2024), and angiogenesis (Li et al., 2024a). Research indicates that YBX1 binds to E2F1 mRNA, forming a positive feedback loop to stabilize and enhance its expression, highlighting the context-dependent regulatory mechanism of YBX1 (Liu et al., 2023a; Liu et al., 2024a). Moreover, YBX1 predominantly interacts with the CDS or 3′UTR regions of target mRNAs, impacting their stability (Liu et al., 2024b; Yu et al., 2023; Zheng et al., 2023b; Huang et al., 2019b). Lin-28 homolog B (LIN28B), recruited by NSUN2, enhances the stability of multiple pathway-related mRNAs, impacting esophageal cancer progression (Su et al., 2021). Despite weaker binding to m5C compared to m6A, YTHDF2 can recognize m5C sites through the Trp432 conserved domain, facilitating rRNA maturation and processing (Dai et al., 2020). RAD52 and FMRP promote mRNA m5C modification at DNA damage sites in a TRDMT1-dependent manner, promoting homologous recombination (Yang et al., 2022c; Chen et al., 2020a).

### m5C writers modification regulates urinary system tumors

The research on RNA modification has revealed the significant impact of m5C modification on urinary system tumors (Table 4). Analysis of NOP2-related data in ccRCC from The Cancer Genome Atlas (TCGA) indicates that NOP2 is highly expressed in renal cancer, showing a significant correlation with poor prognosis. Furthermore, there is an observed relationship between NOP2 expression and MSI, TMB, TNB, and immunity (Wang et al., 2021a). Consistent with the aforementioned bioinformatics analysis findings, NOP2, NSUN4, and NSUN6 exhibit differential high expression levels in renal cancer samples. Additionally, both risk scores and clinicopathological correlations have the potential to serve as valuable prognostic indicators for clear cell renal cell carcinoma (ccRCC) (Li et al., 2021). Furthermore, bioinformatics analysis suggests that NSUN5, a gene prominently expressed in renal cancer, could potentially influence immunity and drug resistance. Recent studies have shown that the suppression of NSUN5 leads to a decrease in cell proliferation, invasion, and migration, while simultaneously increasing levels of apoptosis. The mechanism underlying this phenomenon involves the upregulation of apoptosis-related proteins and activation of the p53 signaling pathway (Li et al., 2023b).

**TABLE 4:** The role of RNA-modifying enzymes in urinary system tumors.

Gene symbol	Enzyme	Cancer type	Role	Main target	Expression	Refs
m5C						
NOP2	Writer	Renal cell cancer	Oncogene	Regulate MSI, TMB, TNB and immunity	Upregulated	Aguilo et al. (2016), Yang et al. (2022b)
NSUN4	Writer	Renal cell cancer	Oncogene	Regulate PI3K-AKT pathway	Upregulated	Aguilo et al. (2016)
NSUN6	Writer	Renal cell cancer	Tumour suppressor	Regulate PI3K-AKT pathway	Downregulated	Aguilo et al. (2016)
NSUN5	Writer	Renal cell cancer	Oncogene	Inhibits the P53 pathway	Upregulated	Fu et al. (2014)
NSUN2, ALYREF	Writer, Reader	Bladder cancer	Oncogene	Enhance oncogenic mRNA splicing and stability	Upregulated	Huber et al. (2015)
NSUN2, YBX1	Writer, Reader	Bladder cancer	Oncogene	Stabilize HDGF3 mRNA	Upregulated	Yang et al. (2022c)
NSUN2, YBX1	Writer, Reader	Prostate cancer	Oncogene	Stabilize AR mRNA	Upregulated	Zhang and Wang (2021)
NSUN5	Writer	Prostate cancer	Oncogene	Stabilize ACC1 mRNA and increases nuclear export	Upregulated	Nulali et al. (2024)
m1A						
TRMT6/61A	Writer	Bladder cancer	Oncogene	Target tRF-3B and ATF6	Upregulated	Wang et al. (2021a), Oerum et al. (2018)
ALKBH3	Eraser	Bladder cancer	Oncogene	Target tweak/Fn14/VEGF	Upregulated	Bhatta et al. (2021)
ALKBH3	Eraser	Prostate cancer	Oncogene	Target MMP9 and AKT	Upregulated	Murakami et al. (2018), Liu et al. (2016)
m7G						
METTL1	Writer	Bladder cancer	Oncogene	Target miR-760, EGFR/EFEMP1 and TROP2	Upregulated	Shimada et al. (2012), Malbec et al. (2019), Cruz and Joseph (2022)
METTL1	Writer	Prostate cancer	Oncogene	Target tRNA and CDK14	Upregulated	Pandolfini et al. (2019), Peter et al. (2017)
eIF4E	Reader	Renal cell cancer	Oncogene	Target activation of multiple oncogenes	Upregulated	Yang et al. (2017), Meng et al. (2024), Nulali et al. (2024)
eIF4E	Reader	Bladder cancer	Oncogene	Target mTOR pathway	Upregulated	Culjkovic et al. (2007), Matsuo et al. (1997), Haimov et al. (2018)
eIF4E	Reader	Prostate cancer	Oncogene	Target PI3K/Akt/mTOR and Ras/MAPK pathway	Upregulated	Amaya Ramirez et al. (2018), Ruscica et al. (2019), Jafarnejad et al. (2018)

Bladder cancer research has shown that NSUN2 co-exists with Aly/REF export factor (ALYREF), contributing to bladder cancer proliferation, invasion, and poor prognosis. Specifically, m5C-mediated ALYREF recognizes RABL6 and TK1 mRNA m5C modification through its K171 domain, enhancing mRNA splicing and stability to increase bladder cancer malignancy (Wang et al., 2023b). Additionally, NSUN2 collaborates with YBX1 to stabilize HDGF3 mRNA by recruiting ELAVL1 and targeting the m5C modification site in the HDGF 3′untranslated region, ultimately promoting bladder cancer progression. Higher expression of m5C key enzymes NUSN2 and YBX1 has been associated with a negative prognosis (Chen et al., 2019a).

The expression of NOP2 is increased in prostate cancer tissue (Sun et al., 2022), potentially promoting the epithelial-to-mesenchymal transition (EMT) and influencing prostate metastasis (Sun et al., 2020b). However, the specific mechanism by which NOP2 mediates m5C regulation in prostate cancer remains unclear. Studies suggest that NSUN2 and androgen receptor (AR) can establish a positive feedback loop in prostate cancer, accelerating disease progression. NSUN2 helps maintain the stability of AR-related mRNA through m5C modification, while ARV7 boosts NSUN2 expression at the transcriptional level (Zhu et al., 2022). NSUN3 and NSUN4 have received less attention in prostate cancer research, but they may play a role in chemotherapy resistance and tumor advancement (Alshaker et al., 2019), potentially serving as risk factors for prostate cancer (Kar et al., 2016). NSUN5 is closely associated with abnormal lipid metabolism in prostate cancer. Research indicates that NSUN5 facilitates the phosphorylation and m5C modification of the Ser327 site on acetyl-CoA carboxylase (ACC1) mRNA. This process recruits ALYREF to bind to ACC1 mRNA, ultimately enhancing its stability and nuclear export. These findings suggest that NSUN5 could be a promising therapeutic target for prostate cancer (Zhang et al., 2023b).

Collectively, these findings expose that m5C-related writers are important for both the regulation and development of urologic tumors and that these key enzymes may be therapeutic targets for urologic tumor therapy.

### m5C readers modification regulates urinary system tumors

ALYREF is highly expressed in bladder cancer and is associated with a poor prognosis (Wang et al., 2023b; Pan et al., 2024). Through its K171 domain, ALYREF recognizes RABL6 and TK1 mRNA m5C modification, promoting their mRNA splicing and stability to increase bladder cancer malignancy (Wang et al., 2023b). Additionally, hypoxia-inducible factor 1A transcriptionally activates ALYREF expression, which then binds to the 3′-untranslated regions of PKM2 mRNA, leading to elevated PKM2 expression. Consequently, PKM2 mediates glycolysis to promote tumor proliferation (Wang et al., 2021b). Another m5C reader, YBX1, is also highly expressed in bladder cancer and is linked to a negative prognosis. Mechanistically, YBX1 recruits ELAVL1 to interact with the m5C site in the 3′untranslated region of HDGF mRNA via the W65 residue in its cold shock domain (Chen et al., 2019c).

In prostate cancer, ACC1 mRNA in the m5C modified state interacts with ALYREF to enhance its stability and facilitate nuclear export, resulting in lipid accumulation and progression of prostate cancer (Zhang et al., 2023b). Prostate cancer-related m5C prognostic model shows that abnormal YBX1 expression affects patient prognosis and immune infiltration (Yu et al., 2022). YBX1 stabilizes ARV7 mRNA in an m5C-dependent manner and forms a positive feedback loop with NSUN2 to promote prostate progression (Zhu et al., 2022).

According to current research findings, ALYREF and YBX1, as m5C readers, play a crucial role in regulating the proliferation, invasion, migration, lipid metabolism, and immune microenvironment of urinary system tumors. Abnormal expression of these proteins is closely associated with prognosis.

## 1-methylcytosine

### m1A overview

Although m1A modification is not as extensive as m6A modification, research from the 1960s has shown its presence in coding RNA and non-coding RNA, such as tRNA, rRNA, and LncRNA (Dunn, 1961; Zhou et al., 2019). This modification alters RNA base pairing, impacting RNA stability and protein translation efficiency, thus regulating molecular biological functions (Wu et al., 2022; Li et al., 2016; Zhou et al., 2016; Kuang et al., 2022). While mRNA m1A modification sites have been less studied, tRNA and rRNA modification sites are more commonly investigated. Advancements in m1A sequencing technology allow for more accurate and precise identification of m1A modification sites. Previous research utilizing m1A antibodies for immunoprecipitation, followed by m1A-ID-seq, has revealed a diverse range of modification sites on both coding and non-coding RNAs. Specifically, mRNA m1A sites have been identified, with a notable enrichment in the 5′untranslated region (Li et al., 2016). However, follow-up analysis showed that previous studies may have had defects such as duplication of detection sites, annotation errors and inaccurate sequencing results, which showed that modification of m1A on mRNA is rare (Schwartz, 2018). Similarly., it was still shown that m1A modification on mRNA is not predominantly enriched in the 5′UTR, and their detection of m1A using m1A antibody at single-nucleotide resolution found that it could be a false-positive result produced by the antibody’s interactions with the m7G-cap, which again gives the idea that m1A modification on mRNA is rare (Grozhik et al., 2019; Khoddami et al., 2019). In the last 2 years, there have been further advances in the study of m1A modification, with the newly developed fluorescence-based RT evolution platform revealing hundreds of m1 - A sites in human multitailed RNAs, as well as modifications of the m1A modification in nascent mitochondrial RNAs (mt-RNAs) (Zhang et al., 2024). As RNA modification detection techniques are improved, more potential sites for m1A modification will be identified, likewise more false modification sites will be excluded.

### Regulation of coding RNAs and non-coding RNAs by m1A

As described earlier, m1A modifications are primarily found in tRNAs, rRNAs, and a small number of mRNAs (Table 1). In eukaryotes, the RNA processing enzyme 8 targets position 645 of 25S rRNA for m1A modification, impacting carbohydrate metabolism and translation (Yang et al., 2023b; Li et al., 2022a; Yip et al., 2013; Peifer et al., 2013; Sharma et al., 2018). Moreover, in humans, the nuclear methyl protein (NML) catalyzes methylation at site 1,322 of 28S rRNA (Peifer et al., 2013). The TRMT61B-mediated m1A modification at position 947 of mitochondrial rRNA plays a crucial role in regulating mitochondria-encoded proteins (Bar-Yaacov et al., 2016). Furthermore, NML-mediated methylation modification of m1A in 28S rRNA enhances the translation of target proteins while suppressing the expression of P53 (Waku et al., 2016). For tRNA, m1A modification promotes partial tRNA methylation in hepatocellular carcinoma, leading to increased translation of PPARδ and impacting cholesterol synthesis (Wang et al., 2021c). Additionally, TRMT6-mediated m1A modification of tRNA negatively regulates gene silencing of tRF-3s (Su et al., 2022). The high m1A modification status of tRNAs in T cells enhances the translation efficiency of MYC-associated proteins (Liu et al., 2022; Li et al., 2024b). In summary, m1A modification promotes tRNA stability and correct folding of tRNA structure. As for mRNA, m1A modification primarily occurs in nuclear mRNA in the 5′UTR (near the translation start codon) and in mitochondrial mRNA in the CDS and 3′UTR (Li et al., 2016; Dominissini et al., 2016). Generally, m1A is situated near translation initiation sites to regulate translation initiation and enhance translation efficiency (Safra et al., 2017). For instance, m1A at the SP100 mRNA initiation site promotes its translation process (G et al., 2024). However, when m1A modification is found in the mRNA 5′UTR and CDS region, it may disrupt base complementary pairing, thereby inhibiting the translation process (Li et al., 2016; Wu et al., 2022; Kuang et al., 2022). Although the modification of non-coding RNAs (LncRNAs, miRNAs, and circRNAs) by m1A is currently less studied, the shared key enzymes with m6A suggest potential expansion in future research.

### Effect of m1A modification on the signal transduction pathways

Current studies have shown that in gastrointestinal tumors, m1A modification may play a regulatory role in the PI3K/AKT pathway, with specific mechanisms requiring further investigation (Zhao et al., 2019; Shi et al., 2020). In patients with advanced hepatocellular carcinoma, higher levels of m1A modification of tRNAs have been associated with increased PPARδ translation efficiency, leading to elevated cholesterol synthesis and activation of the Hedgehog pathway (Yang et al., 2023b). Additionally, during T cell pre-activation, TRMT6 upregulates tRNA m1A58 modification, enhancing the synthesis of MYC-related proteins (Yip et al., 2013). Knockdown of MNL has been found to promote RPL11 expression through m1A modification, resulting in inhibition of the ubiquitinating enzyme MDM2 and subsequent activation of the P53 pathway (Waku et al., 2016). These findings highlight the current research focus on the impact of RNA m1A modifications on signaling pathways (Table 2).

### The m1A writers

m1A modifying enzymes can be categorized into writers, erasers, and readers, which are responsible for modifying various types of RNA (Table 3). Like other types of RNA modifications, m1A modifications have a set of key enzymes including writers, erasers, and readers, of which the writers consist of TRMT6, TRMT61A, TRMT61B, TRMT10C, and NML (Safra et al., 2017; El Yacoubi et al., 2012; Ozanick et al., 2005; Vilardo et al., 2018; Ozanick et al., 2005). Although m1A modification is less prevalent than m6A, it can occur in various types of RNA (Figure 1). Currently known m1A-modified writers mainly belong to the tRNA methyltransferase protein family members, of which the TRMT6/TRMT61A complex is mainly responsible for the formation of stable m1A modifications in tRNAs at the neck-loop region (Li et al., 2017b)and regulation of some mRNAs (Chujo and Suzuki, 2012). Trmt61B is mainly responsible for m1A modification at site 58 in tRNA (Leu (UUR)), tRNA (Lys) and tRNA (Ser(UCN)) within mitochondria (Safra et al., 2017). In addition, it has been found that m1A modification at site 947 of 16S rRNA in mitochondria is also regulated by TRMT61B (Sun et al., 2022). Discrimination of individual base modifications for m1A is lacking, and TRMT10C was developed in mitochondria to specifically catalyze the m1A site, whose catalytic process is tightly regulated (Safra et al., 2017). In addition, TRMT10C in mitochondria is responsible for m1A modification of the mt-tRNA 9 site, which is essential for proper tRNA folding and mitochondrial maturation (Vilardo et al., 2018; Bhatta et al., 2021). Mechanistically, TRMT10C and SDR5C1 form a complex that binds to tRNA conserved sequences thereby promoting m1A modification at this locus (Murakami et al., 2018). NML (also known as yeast homologues RRP8) facilitates 60S ribosomal subunit formation by m1A modification of 28S rRNAs (Sharma et al., 2018; Waku et al., 2016).

### The m1A erasers

The current m1A-associated erasers mainly include AlkB family proteins (ALKBH1 and ALKBH3) and FTO. Although ALKBH1 does not affect mitochondrial structure, its m1A modification of mitochondrial tRNA regulates respiration and protein translation (; Liu et al., 2016) and stimulates the mitochondrial unfolded protein response (Wagner et al., 2019). Furthermore, ALKBH1 in mitochondria dynamically regulates m1A modification of tRNA, decreases tRNA stability and promotes tRNA cleavage (Rashad et al., 2020). Recent studies have shown that m1A modification of mRNA by ALKBH1 promotes colon cancer progression and poor prognosis, which mechanistically may be due to its modification of METTL3 mRNA (Chen et al., 2023a). Possesses a tRNA-binding structural domain similar to ALKBH1, so ALKBH3 also functions to modify tRNAm1A to promote ribosome assembly (Chen et al., 2019c). Similarly, ALKBH3 can regulate mRNA stability and protein translation through m1A modification, which is achieved by binding different regions of the mRNA (Kuang et al., 2022; G et al., 2024; Seo and Kleiner, 2020; Woo and Chambers, 2019). ALKBH3 also eliminates DNA/RNA deregulated methylation modifications, thereby completing the DNA damage repair process (Aas et al., 2003; Ougla et al., 2004). FTO has been widely studied in the modification of m6A, but in the modification of m1A, its substrate selection is more limited, such as the circular structure in tRNA rather than the linear structure, thereby inhibiting the translation process (Wei et al., 2018).

### The m1A readers

YTH structural domain family proteins (YTHDF1-3 and YTHDC1) act as readers of m1A to recognize multiple RNA methylation modifications (Dai et al., 2018). It was shown that YTHDF1 not only promotes the mRNA translation process through m1A dynamic modification (Dunn, 1961; Dai et al., 2018), but also reduces the translation efficiency by forming a complex in concert with eRF1 (Wu et al., 2023), which might be due to the Watson-Crick destructive properties generated by its binding to different m1A modification sites (Safra et al., 2017; Li et al., 2017b). In addition, YTHDF2 and YTHDF3 can interact with some mRNAs to destabilize transcripts through m1A modification. In addition, YTHDF2 and YTHDF3 can interact with some mRNAs to destabilize transcripts through m1A modification (Seo and Kleiner, 2020; Zheng et al., 2020), and Trp432, a conserved residue in YTHDF2, is an important site for recognizing m1A modification (Dai et al., 2018). YTHDC1 is involved in the repair of RNA damage-induced DNA breaks (RDIBs) through m1A modification (Tsao et al., 2024).

### m1A writers modification regulates urinary system tumors

The impact of m1A modification on bladder cancer is gradually being uncovered (Table 4). Research has indicated that increased m1A modification in bladder cancer is closely linked to the abnormal expression of TRMT6/61A, which controls the target mRNA unfolded protein response (UPR) and bladder cancer advancement through tRF-3B (Li et al., 2022a). Specifically, the removal of TRMT6/TRMT61A resulted in decreased mRNA levels of the conventional transcription factor UPR ATF6-associated targets, leading to a reduction in bladder cancer cell proliferation and stress resistance (Monshaugen et al., 2024). Since the role of m1A writers in urologic tumors has not been extensively discussed, further comprehensive studies are necessary.

### m1A erasers modification regulates urinary system tumors

ALKBH3 has been thoroughly researched in the field of bladder cancer. Silencing ALKBH3 results in the halting of tumor cell cycle progression by influencing NOX-2 to increase ROS activity. Moreover, ALKBH3 plays a role in regulating VEGF expression by affecting Tweak and Fn14. However, the exact mechanism by which ALKBH3 controls m1A modification is still not fully understood (Shimada et al., 2012).

ALKBH3 is recognized as a marker for prostate cancer, with its elevated levels being closely linked to the aggressiveness of the cancer and its ability to inhibit apoptosis (Koike et al., 2012; Ueda et al., 2018). Due to the absence of a reliable enzymatic assay for ALKBH3, a novel electrochemical signaling assay was created to study its m1A modification. This assay operates on the principle that the alkyl group of the Fc-DNA probe is eliminated, resulting in the formation of a 3′flat end on the DNA, ultimately leading to a reduction in the signal produced by Fc (Cheng et al., 2022).

## N7-methylguanosine

### m7G overview

Similar to m5C and m1A, m7G is a common modification of RNA, with methylation of the guanine N7 site, which regulates RNA structure by altering molecular charge changes (Boulias and Greer, 2019). The m7G modification can occur within a variety of RNAs, including mRNA 5′capsids (Furuichi, 2015) and some precursor tRNAs, protecting them from stable maturation (Ohira and Suzuki, 2016). This modification functionally regulates mRNA splicing, stabilization and protein synthesis and translation (Furuichi, 2015). In addition, m7G modifications are widely found not only within mRNAs and tRNAs, but also in rRNAs, miRNAs, and lncRNAs, which are responsible for RNA processing, stabilization, and translocation (Zhu et al., 2018; Pandolfini et al., 2019; Zhang et al., 2022; Lu et al., 2023; Dai et al., 2021; Volpon et al., 2016). In the early days, m7G-MeRIP sequencing (MeRIP-seq) was utilized to analyze the m7G modification sites of transcriptomic mRNAs and tRNAs (Zhang et al., 2019). With the development of sequencing technology, it was found by immunoprecipitation sequencing (m7G miCLIP-seq) that the m7G modification is mainly located in the 5′UTR region of mRNAs, and the modification significantly accumulates in the CDS and 3′UTR regions under stress (Malbec et al., 2019). A novel predictor, THRONE, was validated to more accurately predict m7G modification site (Shoombuatong et al., 2022).

### Regulation of coding RNAs and non-coding RNAs by m7G

m7G plays a role in regulating mRNA stability and translation processes (Table 1), with its most probable binding mode against the mRNA cap structure involving the stacking of Trp-102 and hydrogen bond pairing of Glu-105 (Ueda et al., 1991). METTL1 has been shown to enhance VEGFA mRNA translation in a manner dependent on m7G methylation, thereby facilitating tumor progression (Zhao et al., 2021). METTL1 plays a role in promoting CDK11 and ATF5 mRNA stability in an m7G manner, impacting tumor progression (Chen et al., 2023b; Ma et al., 2021). Additionally, RNA modification does not act independently; the METTL3/METTL1-mediated m6A/m7G dual RNA modification boosts TROP2 translation, leading to the promotion of bladder carcinogenesis (Chen et al., 2023b). Furthermore, a codon-frequency-dependent mechanism of m7G-tRNA decoding catalyzed by METTL1 is known to facilitate mRNA translation in various types of tumors (Ma et al., 2021; Dai et al., 2021; Huang et al., 2023; Chen et al., 2022a; Han et al., 2022; Orellana et al., 2021; Wang et al., 2023c; García-Vílchez et al., 2023; Zhao et al., 2024b; Huang et al., 2022). The structural basis of METTL1-mediated tRNA modification involves the αC region of METTL1 becoming helical and stabilizing the tRNA along with the α6 helix. Additionally, phosphorylation of the N-terminal S27 of METTL1 impacts its catalytic activity (Li et al., 2023c). While sequencing results have identified m7G modifications in rRNAs, further research is needed to determine the specific sites of these modifications and their spatial structural changes (Bujnicki and Rychlewski, 2001). miRNAs can be regulated by m7G modifications, which can inhibit the expression of target mRNAs. Research has shown that METTL1-mediated modification of miRNAs by m7G can disrupt the secondary structure of precursor miRNAs. This was observed through borohydride reduction sequencing and RNA immunoprecipitation (Pandolfini et al., 2019). In the context of bladder cancer, METTL1 is involved in m7G modification of miR-760, leading to the suppression of ATF3 mRNA expression (Xie et al., 2022). Additionally, METTL1 plays a role in mediating m7G modification of miR-26a-5p, indirectly inhibiting FTH1 mRNA expression and translational efficiency (He et al., 2024).

### Effect of m7G modification on the signal transduction pathways

Inhibition of m7G modification by knockdown of METTL1 reduces MAPK pathway activity, ultimately inhibiting the proliferative phenotype (Deng et al., 2020; Wang et al., 2024). Likewise, NCBP2-mediated m7G modification promotes c-JUN mRNA translation, activating the MEK/ERK pathway (Xie et al., 2023). Knockdown of METTL1 decreases the level of m7G modification of tRNAs, resulting in the downregulation of oncogenic transcripts and the PI3K/AKT/mTOR pathway (Chen et al., 2022a). In nasopharyngeal carcinoma, METTL1 enhances the efficiency of target mRNA codon recognition during translation by modifying tRNAs with m7G, thereby activating the WNT/β-catenin signaling pathway (Chen et al., 2022b). Quaking proteins (QKIs) recognize internal mRNA m7G modifications and downregulate key targets within the Hippo pathway (Zhao et al., 2023). The modification of tRNA m7G by METTL1 leads to the upregulation of genes associated with the EGFR pathway, a transcript linked to oncogenesis, thereby facilitating cancer advancement and resistance to drugs (Dai et al., 2021; Huang et al., 2023). In summary, RNA m7G modifications play a crucial role in regulating signaling pathways and promoting tumor progression (Table 2).

### The m7G writers

m7G modifying enzymes can be categorized into writers, erasers, and readers, which are responsible for modifying various types of RNA (Table 3). The METTL1/WDR4 complex, a well-researched m7G methyltransferase, has homologs in *Saccharomyces cerevisiae* known as Trm8 and Trm82. These homologs are essential in *S. cerevisiae* for tRNA m7G modification (Figure 1) (Alexandrov et al., 2002). METTL1/WDR4 is responsible for m7G modification of various RNAs, impacting a range of biological functions. The complex directly modifies mRNA m7G, leading to increased mRNA output and translation efficiency. For example, VEGFA and Sptbn2 mRNA m7G modification by METTL1/WDR4 enhances their stability and translation efficiency (Zhao et al., 2021; Li et al., 2023d). METTL1/WDR4 indirectly promotes partial mRNA translation efficiency through high-frequency m7G tRNA decoding codons, impacting stem cell self-renewal, tumor progression, and drug resistance (Ma et al., 2021; Dai et al., 2021; Wang et al., 2023c; Lin et al., 2018). Additionally, METTL1 can individually mediate primary miRNA m7G modifications that influence the stability of let-7e-5p miRNA, miR-760, and miR-26a-5p (Pandolfini et al., 2019; Xie et al., 2022; He et al., 2024). RNMT plays a crucial role in maintaining mRNA cap methylation and, in conjunction with the cofactor RAM, enhances mRNA stabilization and translational efficiency; however, its monomer exhibits a weak affinity for mRNAs (Gonatopoulos-Pournatzis et al., 2011; Trotman et al., 2017; Bueren-Calabuig et al., 2019). The human WBSCR22/TRMT112 complex, similar to its yeast counterpart, is primarily involved in m7G modification of 18s rRNA and ribosome maturation (Figaro et al., 2012; Õunap et al., 2013; Zorbas et al., 2015; Taforeau et al., 2013; Joshi et al., 2005; Carroll and Borden, 2013; Culjkovic et al., 2007; Matsuo et al., 1997; Haimov et al., 2018; Cruz and Joseph, 2022; Peter et al., 2017; Weber et al., 2020; Amaya Ramirez et al., 2018; Ruscica et al., 2019).

### The m7G readers

The study revealed that the m7G-modified readers of mRNAs primarily consist of eIF family members eIF4E1 (eIF4E), eIF4E2 (4EHP), and eIF4E3 (Joshi et al., 2005). The translation initiation factor eIF4E identifies the 7-methylguanosine (m7G) cap structure in the 5′-UTR of mRNA, facilitating mRNA binding to ribosomes and enhancing mRNA transport from the nucleus to the cytoplasm (Carroll and Borden, 2013; Culjkovic et al., 2007). It was noted that the eIF4E monomer alone does not facilitate mRNA translation activity; instead, it must form a complex with eIF4G to recognize the mRNA m7G modification, a process reliant on a curved eight-stranded antiparallel beta sheet (Matsuo et al., 1997; Haimov et al., 2018; Cruz and Joseph, 2022). Early 4EHP was initially identified as a translational repressor that could interact with GIGYF1/2 to collectively regulate mRNA degradation and translation repression (Peter et al., 2017; Weber et al., 2020). Mechanistically, GIGYF proteins recruit CCR4-NOT to conserved mRNA sequences to facilitate mRNA decay and translational repression (Amaya Ramirez et al., 2018; Ruscica et al., 2019). Additionally, 4EHP mediates miRNAs to achieve translational repression through the competing endogenous RNA (ceRNA) theory (Jafarnejad et al., 2018; Zhang et al., 2021). It has been demonstrated that 4EHP and GIGYF can form a translational repression complex with other proteins (Villaescusa et al., 2009; Peter et al., 2019). Additionally, 4EHP has been found to interact with coactivators such as TRS and ARIH1 to enhance the translation process (von Stechow et al., 2015; Jeong et al., 2019; Christie and Igreja, 2023). Compared to eIF4E1, eIF4E3 exhibits stronger inhibition of mRNA translation, export, and tumor suppressor factors (Volpon et al., 2013). eIF4E3 utilizes its residue structure to interact with m7G modification through electrostatic and van der Waals forces (Osborne et al., 2013). The m7G-related reader NCBP2 plays a critical role in identifying m7G modifications for mRNA stabilization and translation processes (Xie et al., 2023). Dysregulation of NCBP2 has been observed in various tumors and is linked to immune responses (Li et al., 2022b; Zhou et al., 2022; Xu et al., 2023; Liu et al., 2023b).

### m7G writers modification regulates urinary system tumors

Reports of m7G modification in urinary system tumors are relatively limited and have far-reaching research significance (Table 4). In the context of bladder cancer, METTL1 has been shown to promote proliferation, invasion, and migration by modifying tRNAs in an m7G-dependent manner, which enhances the translational efficiency of EGFR/EFEMP1 and TROP2 (Chen et al., 2023b; Ying et al., 2021). Furthermore, METTL1 can also modify miR-760 in an m7G-dependent manner to suppress ATF3 mRNA expression, thereby contributing to the progression of bladder cancer (Bujnicki and Rychlewski, 2001). In prostate cancer, METTL1 plays a role in regulating the levels of interferons and immune factors in the immune microenvironment through m7G modification. Specifically, this modification regulates the production of new small non-coding RNAs derived from 5′tRNA fragments (García-Vílchez et al., 2023). The abnormal increase in CDK14 levels in prostate cancer is linked to METTL1-mediated modification of its mRNA expression in an m7G manner, resulting in enhanced CDK14 mRNA stability (Zhang et al., 2023c).

### m7G readers modification regulates urinary system tumors

Disturbed expression of eIF4E in renal cancer may be associated with poor prognosis (Ichiyanagi et al., 2018). Research has shown that eIF4E mediates enhanced translation of proto-oncogenes (Yang et al., 2023c), promotes proliferation and invasion of renal cancer (Li et al., 2017c), and is closely associated with sunitinib resistance (Chen et al., 2020b). However, despite being a classical translation initiation factor, eIF4E has not been extensively discussed in its role of regulating downstream proto-oncogenes through the m7G mechanism.

Phosphorylation modification of the eIF4E protein has been found to promote the progression of bladder cancer (Jana et al., 2021). Conversely, inhibiting eIF4E phosphorylation has been shown to decrease bladder tumor cell activity and proliferation (Kyou Kwon et al., 2014; Chi et al., 2015). Furthermore, eIF4E may play a role in resistance to Pirarubicin by affecting autophagy (Li et al., 2015).

The role of eIF4E in prostate cancer is influenced by factors such as phosphorylation modification and miRNA (D'Abronzo and Ghosh, 2018; Furic et al., 2010; Xu et al., 2021). Dysregulation of eIF4E has been shown to affect proliferation, invasion, migration, and chemotherapy resistance in prostate cancer (Kwegyir-Afful et al., 2016; Liu et al., 2020). Mechanistically, eIF4E facilitates m7G modification of specific mRNA 5′end structures, impacting key pathways in prostate cancer development like PI3K/Akt/mTOR and Ras/MAPK, ultimately regulating the progression of prostate tumors [405]. While eIF4E is extensively studied among m7G-related readers in urologic tumors, the regulation of other molecules in urologic tumors requires further exploration and investigation.

## Conclusion

While m6A is a commonly studied RNA modification (Boccaletto et al., 2022; Chen et al., 2019b), other modifications such as m5C, m1A, and m7G have also been linked to tumor proliferation, invasion, migration, drug resistance, and metabolism (Xue et al., 2023; Wang et al., 2023a; Luo et al., 2022). This review examines the effects of m5C, m1A, and m7G modifications on both coding and non-coding RNAs. It discusses their roles as writers, readers, and erasers in regulating tumor-related signaling pathways, and explores their modulation in the context of urinary tract tumors.

The study of RNA modification is closely tied to advancements in specific antibodies, enhanced sequencing techniques, and more precise algorithms. In the past, due to limitations in sequencing technology, only mass spectrometry could be used to detect RNA modifications that were highly abundant (Boccaletto et al., 2018). However, as the purity of mRNA poly-A samples improved, the study of coding RNA modifications has gained significant attention (Dubin and Taylor, 1975). Various RNA modification sequencing methods and corresponding algorithms each have unique characteristics. Currently, the detection of m5C primarily relies on the sodium bisulfite method or immunoprecipitation with m5C-specific antibodies (García-Vílchez et al., 2019). Immunoprecipitation with specific antibodies can be used to identify enrichment sites of m1A and m7G on RNA (; Zhang et al., 2019). However, sequencing technology has limitations and controversies. For instance, the number of m5C modification sites in animal cells and mouse embryonic stem cells showed significant differences in sequencing results (Squires et al., 2012; Schaefer et al., 2017). Similarly, early sequencing results suggested that m1A was predominantly enriched in the mRNA 5′untranslated region (), but subsequent findings indicated rare m1A enrichment on mRNA (Schwartz, 2018), possibly due to false positive results from m1A antibody detection at single nucleotide resolution (Grozhik et al., 2019; Khoddami et al., 2019). Immunoprecipitation sequencing (m7G miCLIP-seq) revealed changes in m7G modification sites on mRNA under different conditions (Malbec et al., 2019). The development of new prediction tools like THRONE can enhance the accuracy of predicting RNA m7G modifications (Shoombuatong et al., 2022). To achieve more precise enrichment site identification and understand modification mechanisms, further advancements in sequencing technology and algorithm adjustments are necessary.

Both coding and non-coding RNAs can undergo modifications such as m5C, m1A, and m7G to either stabilize the RNA or enhance translation levels. For instance, modifications like m5C, m1A, and m7G in tRNAs play a role in bolstering their structural integrity and improving protein translation efficiency, impacting proto-oncogene oncogenicity (Li et al., 2023a), cellular differentiation processes (Van Haute et al., 2019), cholesterol synthesis (Wang et al., 2021c), the immune microenvironment (Liu et al., 2022; Li et al., 2024b), and ROS levels (Li et al., 2023c). Interestingly, recent findings suggest that reducing m5C levels in tRNAs may lead to dysfunction in tRNAGly coencoder and decreased protein translation efficiency, contradicting previous observations (Blaze et al., 2021). The m5C and m1A modifications of rRNA play a crucial role in ribosome assembly and protein translation (Metodiev et al., 2014; Waku et al., 2016), impacting intracellular sugar metabolism (Peifer et al., 2013; Sharma et al., 2018) and p53 pathway activity (Waku et al., 2016). Further research is needed to investigate the significance of m7GrRNA modification sites and spatial structure changes (Bujnicki and Rychlewski, 2001). While LC-MS/MS nucleoside analysis and LSTH have identified fewer m5C-modified sites on miRNAs (Lü et al., 2024), studies have shown that m5C modification of LncRNAs (NKILA) and miRNAs (miR-571) can enhance their stability, promoting the progression of cholangiocarcinomas and colon cancers (Zheng et al., 2022; Hou et al., 2024). METTL1-mediated m7G modification of miR-760 and miR-26a-5p has been shown to increase miRNA stability (Xie et al., 2022; He et al., 2024). Furthermore, this modification disrupts the secondary structure of precursor miRNAs, as evidenced by borohydride-reduced sequencing and RNA immunoprecipitation assays (Pandolfini et al., 2019). Generally, m5C, m1A, and m7G modifications are known to enhance mRNA stability and translational efficiency (Chen et al., 2019a; Yang et al., 2022a; Safra et al., 2017; Ueda et al., 1991; Zhao et al., 2021; Chen et al., 2023b; Ma et al., 2021). However, enrichment of specific modification sites can lead to translation termination or reduced mRNA stability. For instance, NSUN6-mediated m5C modification of target genes can trigger translation termination, potentially contributing to quality control of translation fidelity (Selmi et al., 2021). Moreover, when m1A modification occurs in the 5′UTR and CDS regions of mRNA, it may impede translation by disrupting base complementary pairing (Wu et al., 2022; Kuang et al., 2022). In conclusion, the diverse effects of RNA modifications are influenced by the specific sites of modification and resultant changes in RNA and protein structures, highlighting the need for in-depth mechanistic studies.

The regulation of urological tumors by key modifying enzymes of m5C, m1A, and m7G (writers, erasers, and readers) is a significant area of study. Aberrant expression of a variety of RNA modifying enzymes in urological tumors is closely linked to poor prognosis (Chen et al., 2019c; Li et al., 2021; Sun et al., 2022; Koike et al., 2012; Ueda et al., 2018; Ichiyanagi et al., 2018). These RNA modifications play crucial roles in the proliferation, apoptosis, invasion, migration, immune escape, and drug resistance of urological tumors (Li et al., 2023b; Alshaker et al., 2019; Monshaugen et al., 2024; Yang et al., 2023c; Li et al., 2017c; Chen et al., 2020b). As such, these key enzymes hold promise as both diagnostic markers and therapeutic targets for urological tumors. The development of targeted drugs aimed at these key enzymes is expected to play a vital role in the treatment of urological tumors.
